# Advancing patient care with AI: a unified framework for medical image segmentation using transfer learning and hybrid feature extraction

**DOI:** 10.3389/fmed.2025.1589587

**Published:** 2025-07-16

**Authors:** Nazife Çevik, Taner Çevik, Onur Osman, Shtwai Alsubai, Jawad Rasheed

**Affiliations:** ^1^Department of Computer Engineering, Istanbul Arel University, Istanbul, Türkiye; ^2^Department of Computer Engineering, Istanbul Rumeli University, Istanbul, Türkiye; ^3^Department of Electrical and Electronics Engineering, Engineering Faculty, Istanbul Topkapi University, Istanbul, Türkiye; ^4^Department of Computer Science, College of Computer Engineering and Sciences in Al-Kharj, Prince Sattam Bin Abdulaziz University, Al-Kharj, Saudi Arabia; ^5^Department of Computer Engineering, Istanbul Sabahattin Zaim University, Istanbul, Türkiye; ^6^Department of Software Engineering, Istanbul Nisantasi University, Istanbul, Türkiye; ^7^Applied Science Research Center, Applied Science Private University, Amman, Jordan

**Keywords:** intestinal polyps, brain tumors, deep learning, local binary patterns, gray-level co-occurrence matrix

## Abstract

**Background:**

Accurate medical image segmentation significantly impacts patient outcomes, especially in diseases such as skin cancer, intestinal polyps, and brain tumors. While deep learning methods have shown promise, their performance often varies across datasets and modalities. Combining advanced segmentation techniques with traditional feature extraction approaches may enhance robustness and generalizability.

**Objective:**

This study aims to develop an integrated framework combining segmentation, advanced feature extraction, and transfer learning to enhance segmentation accuracy across diverse medical imaging (MI) datasets, thus improving classification accuracy and generalization capabilities.

**Methods:**

We employed independently trained U-Net models to segment skin cancer, polyps, and brain tumor regions from three separate MI datasets (HAM10000, Kvasir-SEG, and Figshare Brain Tumor dataset). Moreover, the study applied classical texture-based feature extraction methods, namely Local Binary Patterns (LBP) and Gray-Level Co-occurrence Matrix (GLCM), processing each Red Green Blue (RGB) channel separately using an offset [0 1] and recombining them to create comprehensive texture descriptors. These segmented images and extracted features were subsequently fine-tuned pre-trained transfer learning models. We also assessed the combined performance on an integrated dataset comprising all three modalities. Classification was performed using Support Vector Machines (SVM), and results were evaluated based on accuracy, recall (sensitivity), specificity, and the F-measure, alongside bias-variance analysis for model generalization capability.

**Results:**

U-Net segmentation achieved high accuracy across datasets, with particularly notable results for polyps (98.00%) and brain tumors (99.66%). LBP consistently showed superior performance, especially in skin cancer and polyp datasets, achieving up to 98.80% accuracy. Transfer learning improved segmentation accuracy and generalizability, particularly evident in skin cancer (85.39%) and brain tumor (99.13%) datasets. When datasets were combined, the proposed methods achieved high generalization capability, with the U-Net model achieving 95.20% accuracy. After segmenting the lesion regions using U-Net, LBP features were extracted and classified using an SVM model, achieving 99.22% classification accuracy on the combined dataset (skin, polyp, and brain).

**Conclusion:**

Integrating deep learning-based segmentation (U-Net), classical feature extraction techniques (GLCM and LBP), and transfer learning significantly enhanced the accuracy and generalization capabilities across multiple MI datasets. The methodology provides robust, versatile framework applicable to various MI tasks, supporting advancements in diagnostic precision and clinical decision-making.

## 1 Introduction

The incidence of cancer worldwide has remained high in recent years. Additionally, each year, tens of millions of people receive a new cancer diagnosis. Meanwhile, different forms of cancer kill millions to almost tens of millions of people (1). According to the WHO, cancer will be the top cause of death globally in 2020, taking around 10 million lives (2). When it came to new cancer cases in 2020, the most prevalent were 2.26 million cases in the breast, 2.21 million in the lung, 1.93 million in the colon and rectum, 1.41 million in the prostate, 1.20 million in the skin (non-melanoma), and 1.09 million in the stomach. Pathology and imaging diagnostics are the primary methods used to diagnose cancer (3, 4). Increasing the survival percentage of cancer patients requires early detection (5), and effective and non-invasive early screening has emerged as a crucial study area. Magnetic resonance imaging (MRI), computed tomography (CT), X-rays, B-ultrasound, and others are examples of imaging techniques (6). Since an MRI scan can differentiate between different types of tissues, it can help spot cancer in different parts of the body (7). Medical image segmentation allows researchers and doctors to precisely identify and examine particular structures by dividing a medical image into discrete regions of interest. This segmentation procedure is important since thorough and precise evaluations are critical to patient care in radiology, pathology, and other medical specialties. Completing the regional segmentation’s nodules and tracheal placement area is challenging (8). Screening and symptomatic disease management are the foundations of imaging’s involvement in cancer management. Imaging will be used in cancer treatment in the future for targeted, minimally invasive, and pre-symptomatic treatments (9). Image guidance will be used to develop locally activated medication delivery and less invasive targeted therapy (10–14). Because tissue and fluids in the body absorb and scatter light, clinical optical imaging has mostly been restricted to endoscopic, catheter-based, and superficial imaging strategies. Since cancer is a complex disease, imaging must be able to show the many pathophysiological phases and mechanisms. Combining independent and uncorrelated imaging technologies will result in diagnostic orthogonality by employing diverse modalities, imaging agents, and biomarkers in general. Diagnostic imaging agents delivered intravenously, intra-arterially, or through natural orifices will become more prevalent in cancer imaging (15–17). Medical image segmentation aims to identify anatomical features in medical images, such as organs, lesions, tissues, etc. Many clinical applications depend on this basic phase, including computer-aided diagnosis, therapy planning, and illness progression tracking (18, 19). Precise segmentation can yield trustworthy target structure volumetric and morphological data, supporting numerous therapeutic uses such as quantitative analysis, surgical planning, and illness detection (20–22). Artificial intelligence (AI), particularly deep learning methods, has become a potent tool for improving and automating image segmentation in recent years. Medical image processing and analysis have seen tremendous success with deep learning algorithms, particularly Convolutional Neural Networks (CNNs), which provide quicker, more accurate, and repeatable results than manual techniques. Large annotated datasets can be used to train these models, enabling AI systems to identify intricate patterns and structures in medical images and provide accurate segmentation with little human assistance (23). CNN-based techniques can automatically extract the most valuable characteristics from massive datasets for medical segmentation. To improve diagnostic efficiency and make medical images more comprehensible, the initial and crucial stage in the analysis of medical images is medical image segmentation (24). To help doctors create more accurate diagnoses, we must segment the areas of medical images we focus on and extract pertinent features. This will give a solid foundation for clinical diagnosis and pathology research. Semantic segmentation, or the recognition of images at the pixel level, is typically referred to as image segmentation in deep learning. Semantic segmentation finds groups of pixels and categorizes them based on several attributes. Semantic segmentation research typically uses transfer learning. With transfer learning, a model already trained on a sizable dataset can be modified for a new job by teaching it to recognize general features. This is accomplished by retraining only the final layers of the model and freezing the other layers. As a result, the model retains the knowledge it gained from the prior task while adjusting to the inputs in the new one. Limited datasets and the inability to directly access current literature from another topic are two scenarios where transfer learning is used to help. Transfer learning has been effective in several applications, including text classification (25), satellite image segmentation (26), facial expression identification (27), and more.

Transfer learning offers an effective method to solve complex image analysis problems using the power of deep networks. However, classical feature extraction methods that can form the basis of transfer learning algorithms are also important in some cases. Traditional methods, such as the Gray-Level Co-Occurrence Matrix (GLCM) and the Local Binary Pattern (LBP), can create meaningful inputs for transfer learning models or provide complementary information in fine-tuning the models. Thus, combining classical and modern techniques allows obtaining powerful results, especially in limited datasets. In this context, GLCM and LBP are two approaches that stand out from traditional image processing techniques. GLCM is a method that models the spatial relationships of pixel pairs at grayscale levels to examine the textural properties of an image. The use of GLCM features in medical image analysis has rapidly expanded in recent years. Examples include the analysis of MRI and ultrasound images of the liver (28, 29), the heart (30), X-ray mammography (31, 32), breast cancer (33, 34), prostate cancer (35–37), and brain cancer (38–40). Haralick et al. (41) proposed a general process for determining the textural characteristics of image blocks. The texture’s statistical nature is considered while calculating features in the spatial domain. Mall et al. (42) used machine learning techniques to divide the MURA (musculoskeletal radiographs) dataset’s bone X-ray images into two categories: those with fractures and those without GLCM features. In the study proposed by Pooja et al. (43), GLCM, LBP, and the Histogram of Oriented Gradient (HOG) are used for feature extraction. The correlation filter method and wrapper-based techniques detect and categorize polyps. On the other hand, LBP creates a histogram by evaluating the intensity differences between neighboring pixels to capture local textural information. During the feature extraction, Shamna and Musthafa (44) suggested HoG and Local Ternary Pattern (LTP). Additionally, the Deep Convolutional Neural Network (D-CNN) was used to fuse the gathered features before forwarding them to the Region-based Convolutional Neural Network to detect many objects. Bhattarai et al. (45) suggested an unsupervised approach to create the pseudo-labels employing HOGs. They learned the deep network’s parameters to minimize the loss of the primary and auxiliary tasks, using pseudo-labels for the auxiliary task and ground truth semantic segmentation masks. The study by (46) extracts the dynamic texture elements of 3D MRI brain images using HOG features to detect Alzheimer’s disease. Another approach proposed a model that uses neural characteristics from MRI images based on HOG to detect brain malignancies (47).

The application of techniques like transfer learning and deep learning in the field of medical image analysis has grown dramatically in recent years. A crucial factor that directly impacts the effectiveness of treatment for many conditions is early identification and accurate classification, particularly for skin cancer, intestinal polyps, and brain tumors. Accurate and precise segmentation is crucial in these imaging difficulties to enhance clinical procedures and improve patient outcomes. However, most current approaches lack generalizability and concentrate on a specific dataset or a restricted feature extraction technique. By working with several datasets and combining transfer learning and sophisticated feature extraction methods, our goal in this study was to improve segmentation performance. In the literature, various medical imaging issues—such as brain tumors, polyps, and skin cancer—are typically treated independently and with diverse techniques. However, this study aims to illustrate how the created technology may be used in various medical imaging situations and to provide a bridge between them. Although the suggested method successfully applies the transfer learning approach to the information transfer of pre-trained models, it combines deep features with statistical approaches, such as GLCM and LBP, as feature extraction techniques to produce more discriminative and meaningful features. This novel combination is anticipated to be highly generalizable to other medical imaging issues. The main contributions of this study are:

- A generalizable method for multiple medical imaging problems is proposed.
- It has been shown that combining transfer learning and classical feature extraction techniques can improve segmentation performance.
- The generalization capacity of the developed model was tested on different datasets.

This article introduces a potential approach for segmenting brain tumors, skin cancer, and polyps to provide a different perspective. Several pre-trained deep learning models, including VGG16, have been tested on various medical datasets, including brain tumors, polyps, and skin cancer. This offers a thorough examination to assess the methodologies’ ability to generalize. Deep learning-based segmentation techniques were used with GLCM and LBP to produce feature sets that were more potent and discriminative. It has been demonstrated that this combination enhances post-segmentation classification performance. This study assessed the overall performance of the suggested approaches using datasets gathered from various anatomical locations and imaging techniques, in contrast to studies in the literature that are often carried out on a single dataset. The suggested method offers integrity in both segmentation and post-segmentation classification performance. Accuracy and time savings are benefits of this functionality, particularly in therapeutic settings. A broad framework that can be applied to clinical diagnosis is suggested by using the same approach to other imaging issues, such as brain tumors, intestinal polyps, and skin cancer.

Rather than proposing a new algorithm, our objective is to design a modular and generalizable pipeline using established techniques (U-Net, LBP, GLCM, and VGG16) to facilitate practical and accurate medical image analysis across diverse domains. Recently, the studies by (48, 49) explored hybrid methods combining segmentation and handcrafted features in biomedical image analysis. Thus, our framework expands on this by integrating these elements into a unified system applicable across multiple datasets.

The remainder of this article is organized as follows. Section 2 details the methodology, including a description of the datasets, the segmentation methods (using U-Net and transfer learning-based approaches), the feature extraction techniques (GLCM and LBP), and the classification strategy employed. In Section 3, we present experimental results, providing quantitative segmentation performance metrics for each dataset (skin cancer, polyps, and brain tumors) and for a combined dataset to evaluate generalization capabilities. Section 4 offers an in-depth discussion of the findings, highlighting the impact of different feature extraction methods, the role of transfer learning, and our approach’s strengths and limitations. Finally, Section 5 concludes the article by summarizing our contributions, discussing potential limitations, and suggesting directions for future research.

## 2 Methodology

Rather than proposing a new algorithm, our objective is to design a modular and generalizable pipeline using established techniques for practical medical image analysis.

### 2.1 Dataset

This study examined a variety of datasets and concentrated on the segmentation of brain tumors, intestinal polyps, and skin cancer. Every dataset was chosen from well-used sources within the pertinent problem domain, and thorough pretreatment procedures were used. The following is a summary of the features of the datasets that were used:

Open-source databases are often used in the literature, and unique datasets gathered as part of specific studies comprise the datasets utilized. Each dataset underwent a thorough examination considering the overall number of samples and the image resolution. To increase segmentation accuracy, masks with images are manually or automatically labeled. Skin Cancer: The HAM10000 database is used to study skin cancer (50). Since the segmentation masks provided by (50) were absent from the original HAM10000 dataset, we used the source data generated by (50). The Figshare Brain Tumor dataset (51) is used for brain tumor segmentation and contains 3,064 pairs of MRI brain images and their mask indicators. In contrast, the Kvasir-SEG database, which includes 1,000 polyp images and the corresponding ground truth from the Kvasir Dataset v2 (52), is used for intestinal polyps. The total number of samples is shown in Table 1.

**Table 1:** Total number of samples in the dataset.

Dataset	Number of samples *(%80 for training, %20 for test)*
Polyp	1,000 (128 × 128)
Skin cancer	10,015 (128 × 128)
Brain tumor	3,064 (128 × 128)

Since the images in the dataset of this study varied in size and dynamic range, it was unsuitable for direct model training. Resizing and normalization procedures were implemented to give the dataset a uniform structure. To match image proportions with the model input, all photos were scaled to 128 × 128. This procedure provided data resized to match the input dimensions required by the network, while optimizing the training process’s computational cost. Additionally, images’ pixel values typically range from 0 to 255. The normalization technique guaranteed faster convergence and kept the model from struggling to learn the significant disparities between these values. To get all pixel values in the range of 0–1, they are divided by 255. This procedure improved learning stability and allowed the model to assign equal weight to each image. These two preprocessing processes improved the model’s performance during training by guaranteeing that the dataset had a more uniform structure.

Since the public datasets lack detailed metadata about acquisition centers or clinical environments, we did not perform external validation. Training and testing were carried out within each dataset. Cross-dataset or multi-institutional generalization is left for future investigation.

To ensure a fair evaluation and avoid data leakage, 10% of the training set was used as a validation set for hyperparameter tuning. The test set was not accessed during training or parameter optimization. Key hyperparameters (such as learning rate, batch size, and number of epochs) were selected based on performance on the validation set. No test data was used during model selection or tuning.

### 2.2 Segmentation method

The U-Net model and the transfer learning-based VGG16 model were the two approaches for image segmentation that were compared in this study. The U-Net model, a convolutional neural network (CNN) structure designed specifically for segmentation challenges, was employed. U-Net, a semantic segmentation technique, was initially proposed for medical image segmentation. Ronneberger et al. (53) debuted U-Net. U-Net’s encoder-decoder architecture is symmetric. The decoder part creates a segmentation mask in the original dimensions using the information taken from the image by the encoder part. The U-Net model was selected because it can learn the details of segmentation masks with high accuracy and generate respectable results even with small datasets. However, the shortcomings of the U-Net model, such as the need for large datasets and the lengthy learning process, are only considered when the model is built from the ground up. As a result, the transfer learning approach was used in the study’s second phase. VGG16, a pre-trained model, was employed in the transfer learning stage. Being a deep network trained on huge datasets (like ImageNet), VGG16 is adept at picking up low-level characteristics (such as edges and textures). To generate a segmentation mask, a decoder section modeled after the U-Net model was added to the encoder portion of the VGG16 model, which was used to extract features from images. This structure made better performance with less data possible, which also speed up the training process through transfer learning.

The parameters of 15 epochs and a batch size of 16 utilized for the training procedure were chosen to balance the model’s performance and training duration. Using the epoch number, 15 was selected as the number of times the model will be trained on all the training data. An adequate learning process is typically achieved by running through the data 15 times during training, especially for small datasets. Choosing too many epochs can lead to overfitting when the model performs well on training data but poorly on new data. The batch size, which is 16, is the quantity of data input into the model concurrently during each training phase. A batch size of 16 ensures training uniformity and optimizes processing time. With a smaller batch, the model can update its parameters more often but may consume more memory. A batch size of 32 is frequently used in various machine-learning situations and is usually a well-rounded choice. The model’s complexity and the amount of data were considered when choosing the study’s parameters. For example, while working with 128 × 128 images, a large batch size number slows the training process—batch size 16 improved memory management. The epoch number 15 was selected to ensure that the model reaches a point during training where accuracy and loss values may stabilize.

While resizing may risk losing important structural details, especially in fine-grained segmentation tasks, we selected 128 × 128 resolution to balance accuracy and computational efficiency. To assess potential performance loss, a subset of polyp and skin images was also resized to 256 × 256, and models were retrained. The difference in accuracy was below 1.2% on average, while computational requirements increased notably. Therefore, we proceeded with a 128 × 128 resolution for all datasets.

### 2.3 Feature extraction

This study’s skin, polyp, and brain datasets sustained different segmentation processes before the GLCM and LBP techniques, unique to each dataset, were used. Segmentation was done using a different U-Net model for every dataset. The goal is to identify various structures in every dataset in a more precise way.

For the GLCM analysis, offset [0 1] was used. The distance and angle that specify the relationship between pixels are referred to as this parameter. After being retrieved independently, the red, green, and blue channels were merged and examined as a single image. Each image’s texture characteristics were extracted using pixel points and radius values for the LBP approach. RGB channels are processed independently and then mixed, as in GLCM. Transfer learning techniques were performed on each dataset independently based on the segmentation outcomes. As a result, GLCM, LBP, and segmentation model performances were contrasted.

In the last step, all datasets were merged to produce a larger and more varied data collection. The following techniques were used successively on this combined dataset: LBP (by separating RGB channels), GLCM [offset (0 1)], and segmentation (U-Net). This procedure was carried out to assess how well the methodologies applied to various datasets.

Texture-based feature extraction techniques such as GLCM and LBP have been employed, particularly in the textural analysis of sections following segmentation. These techniques included modeling textural changes between datasets, classifying the areas produced after segmentation, and integrating with transfer learning models to improve segmentation accuracy. To assess the methods’ generalizability, the analyses carried out independently for each dataset were finished using the combined dataset; consequently, a thorough comparison of the models’ and methodologies’ performances was made.

Feature-level fusion was implemented by concatenating deep features from CNNs and handcrafted features (GLCM and LBP) after extraction. No joint training or architectural integration was performed. This separation allows for interpretability but limits end-to-end learning potential.

Segmentation performance was evaluated using Dice coefficient, IoU (Intersection over Union), accuracy, recall, and specificity. Dice and IoU are especially suited for pixel-wise overlap assessment and are widely accepted in biomedical segmentation tasks.

### 2.4 Classification

In this study, the Support Vector Machines (SVMs) algorithm was preferred to classify the image data after the completed segmentation process. SVM is a method known for its high accuracy rates and generalization abilities and is a frequently used technique, especially in classification problems. The classification process was started using the features obtained from segmentation (such as GLCM and LBP). The features extracted after segmentation were used as input data to the SVM algorithm. We used an SVM classifier due to its proven reliability in handling small feature vectors and its ability to integrate heterogeneous features. However, we recognize that end-to-end deep learning classifiers such as fully connected neural networks or attention-based modules could offer better performance and are considered for future work. SVM works with appropriate kernel functions to create linear or non-linear separation regions. This study used the RBF (Radial Basis Function) kernel function depending on the data distribution. The model was optimized on the training dataset, and its performance was evaluated on the test dataset.

- Accuracy: It served as a fundamental performance metric by computing the proportion of samples the model properly classified among all samples. However, when there is an imbalance between classes, precision is insufficient.
- F-Measure: Calculated as the harmonic mean of the Precision and Recall measures, this metric was intended to show the model’s success in both positive and negative classes and to assess the classification performance in a balanced manner.
- Bias-Variance Composition: The model’s generalization performance was assessed using bias-variance analysis. The mistake happens when the model cannot comprehend the intricate structure present in the training data. Excessive bias causes oversimplification and impairs the model’s accuracy. The bias component indicates the average accuracy of the model across all possible training sets. The variance component indicates how responsive the learning algorithm is to minor modifications in the training set (54).
- Variance: a circumstance in which the model performs poorly on the test data because it has learned too much from the training data. A high variance indicates an overfitting issue.

A thorough assessment of the classification algorithm’s accuracy and generalizability was made possible by complementing performance measures. Bias-variance analysis was essential in comprehending the trade-off between the model’s accuracy and generalization performance, even though the F-measure lessens the effect of class imbalances. This thorough assessment sought to improve the model’s generalization ability and achieve high classification accuracy. Consequently, the SVM algorithm’s classification following segmentation was assessed using carefully chosen metrics, and relevant analyses were conducted to maximize the model’s overall performance. This method improved the dependability and efficiency of the categorization process.

**Figure 1:**
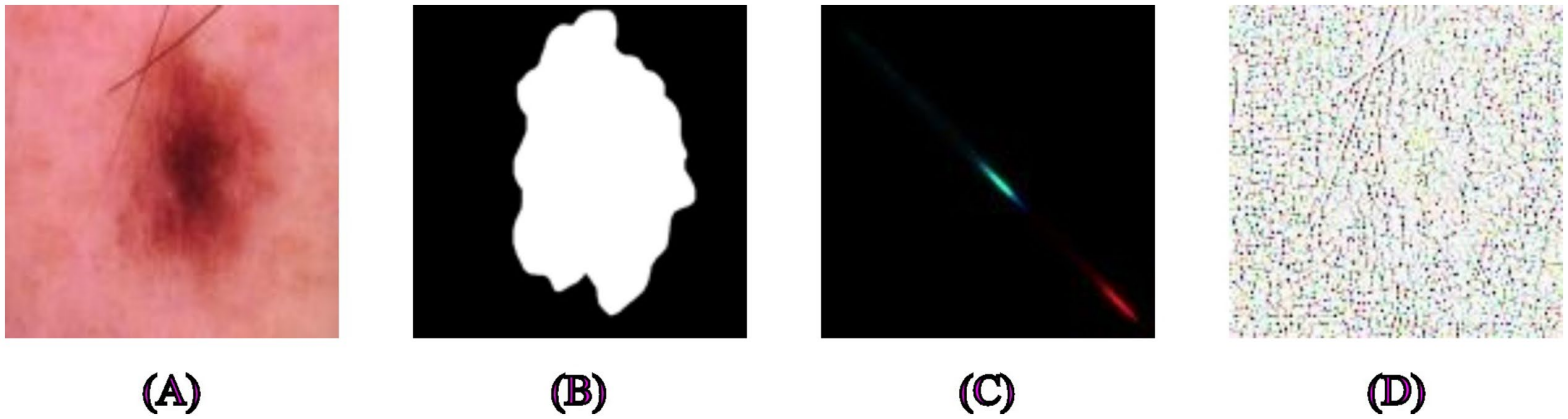
Examples of segmentation and feature extraction on skin cancer images. **(A)** Original skin lesion image from the HAM10000 dataset, **(B)** ground truth segmentation mask, **(C)** corresponding texture-enhanced image obtained by applying Gray-Level Co-occurrence Matrix feature extraction, highlighting spatial relationships between pixels, **(D)** Local Binary Pattern extracted features emphasizing detailed local textural patterns relevant to skin lesion characterization.

**Figure 2:**
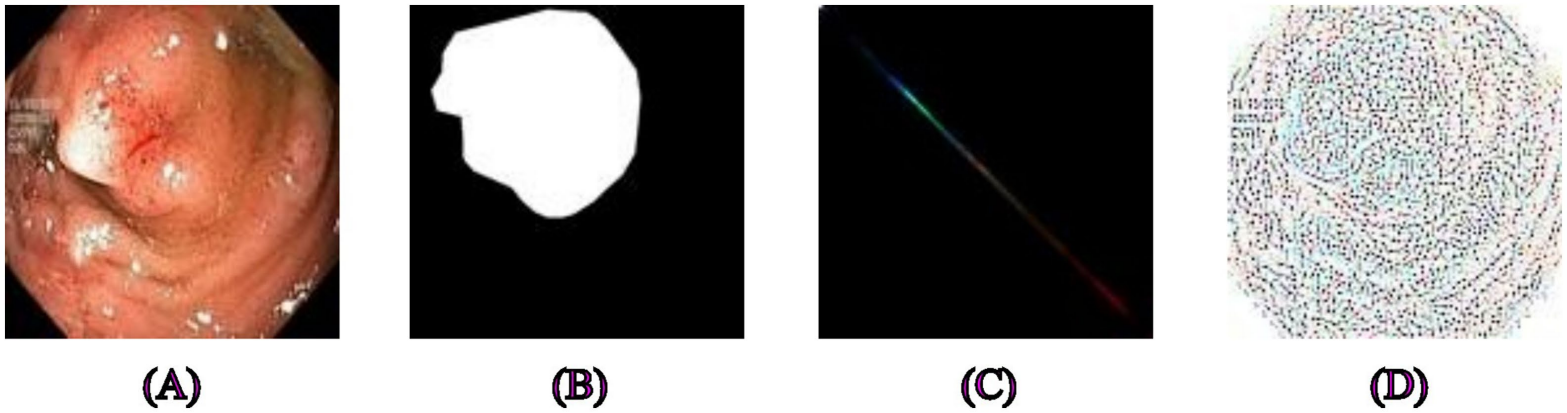
Examples of segmentation and feature extraction on polyp images. **(A)** Original polyp images from the Kvasir-SEG dataset, **(B)** the corresponding segmentation masks. **(C)** Image after applying Gray-Level Co-occurrence Matrix feature extraction, emphasizing textures critical for distinguishing polyps from surrounding tissues, **(D)** Local Binary Pattern-extracted image highlighting local intensity variations that provide robust texture descriptors for precise segmentation.

**Figure 3:**
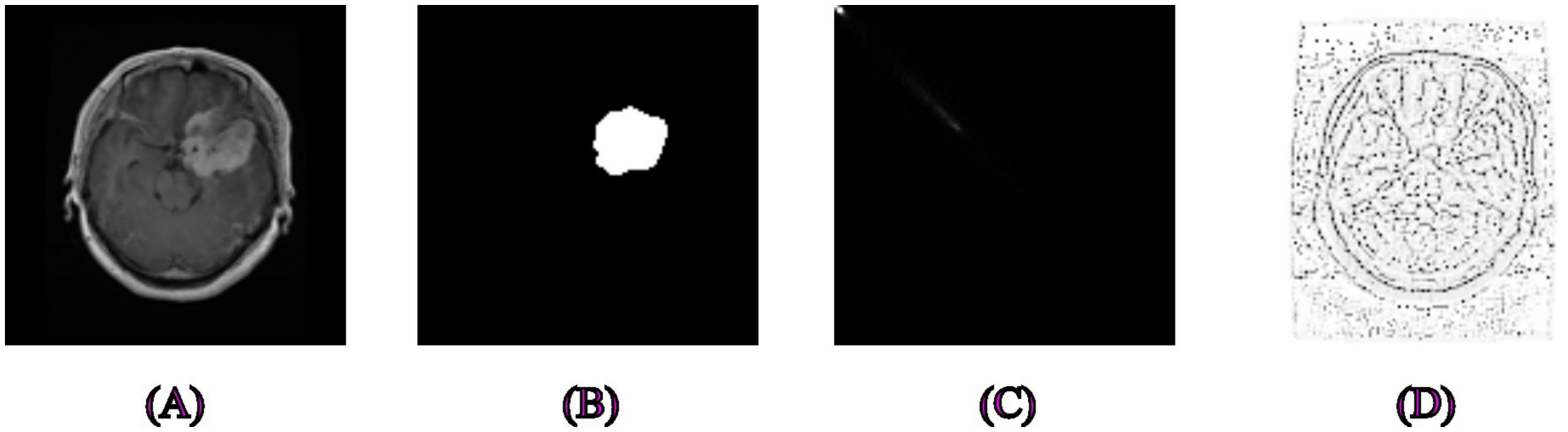
Examples of segmentation and feature extraction on brain tumor MRI images. **(A)** Original brain MRI images from the Figshare dataset, **(B)** Associated ground truth segmentation masks, **(C)** Image processed using Gray-Level Co-occurrence Matrix capturing texture variations to differentiate tumor tissues effectively, **(D)** Local Binary Pattern-extracted image showcasing local texture differences crucial for accurate brain tumor delineation.

First of all, GLCM and LBP feature extraction was done separately for all skin, polyp, and brain tumor datasets, and they are shown in their original form in Figures 1–3. We examined the textural relationships in the image and determined the spatial correlations between pixels in specific orientations (0° in our case) by extracting GLCM features. We evaluated the intensity differences between pixels and their neighbors to analyze the image’s microtextures using LBP feature extraction. We specifically looked at the surface textures of skin lesions and polyps.

**Figure 4:**
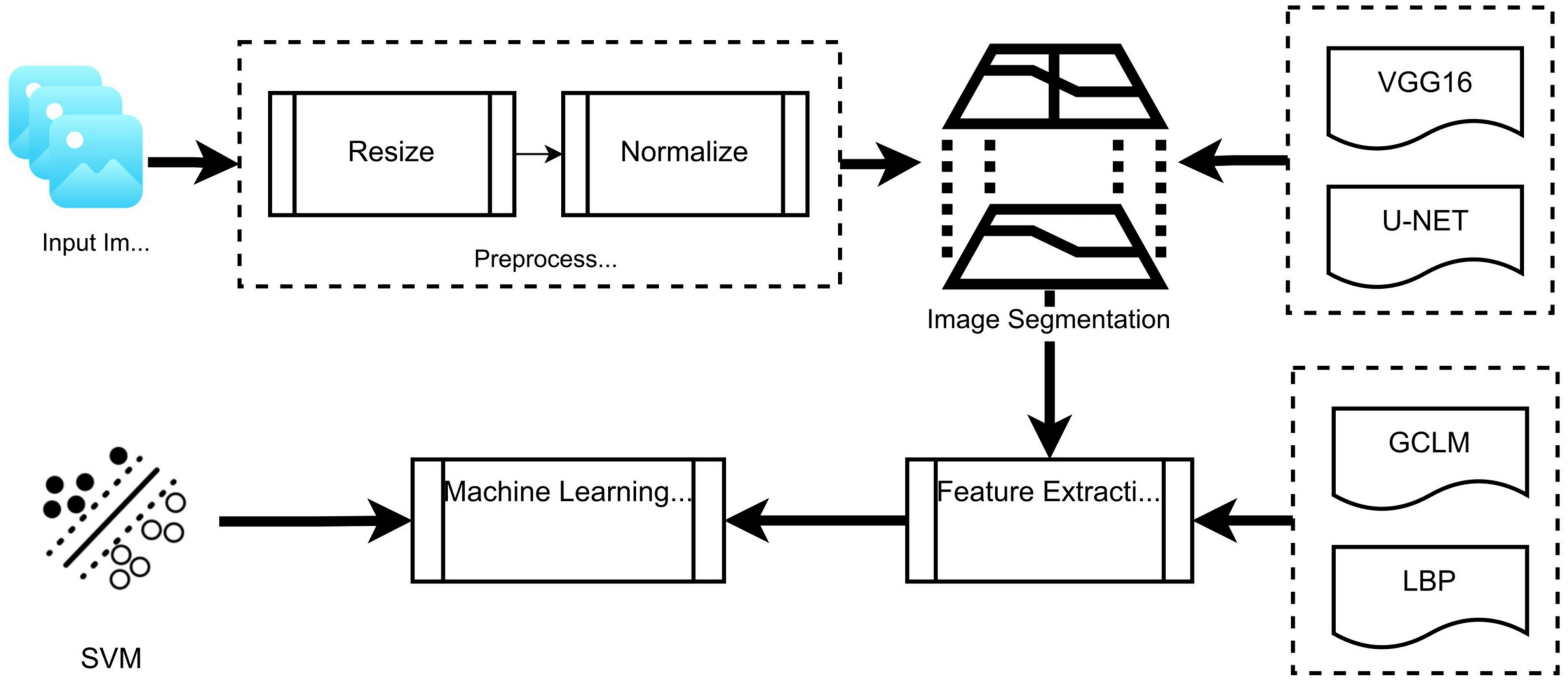
Schematic overview of the proposed framework: From image preprocessing through segmentation (U-Net/VGG16), followed by feature extraction (LBP and GLCM), and final classification using SVM.

The overall workflow of the proposed segmentation and classification framework is illustrated in Figure 4. It includes stages, such as image preprocessing (resizing and normalization), segmentation using U-Net or VGG16-based transfer learning, feature extraction using LBP and GLCM, and final classification using SVM. This schematic is provided to enhance understanding of the integration of traditional and deep learning methods.

### 2.5 Data augmentation strategy

To improve the model’s generalization and reduce overfitting, several augmentation techniques were applied during training. These transformations were randomly applied to each training image during every epoch, using a stochastic pipeline. The following techniques were employed:

- Rotation: Randomly rotating images within a ± 20° range.
- Flipping: Random horizontal and vertical flips.
- Zooming: Scaling the image randomly within a factor of 0.8 to 1.2.
- Translation: Shifting images up to 10% along both axes.
- Brightness/Contrast Adjustment: Slight variations were applied to mimic acquisition differences.

These augmentations increase the diversity of the training data, making the model more robust to variation in position, illumination, and shape. The augmentation was applied on-the-fly during training using stochastic transformations, ensuring that each epoch was exposed to new variations.

The datasets vary significantly in size (e.g., skin: 10,015 vs. polyp: 1,000). To mitigate imbalance and overfitting, we applied data augmentation techniques such as random flipping (horizontal/vertical), rotation, and scaling. These were applied more extensively to smaller datasets to increase effective training diversity.

### 2.6 Bias and variance estimation

To assess the generalization performance of the models, we estimated bias and variance using ensemble-based approximations over multiple runs (*n* = 5). The formulation is as follows (55):

Let $y_{i}$ be the true label of the i^th^ instance, and let ${\hat{y}}_{i}^{\left(j\right)}$ denote the predicted output of the model in the j^th^ run. Then,

- Bias measures the average squared difference between the mean prediction and the ground truth:
$$\text{Bias}=\frac{1}{N}\;\sum_{i=1}^{N}{\left(\frac{1}{n}\sum_{j=1}^{n}{\hat{y}}_{i}^{\left(j\right)}-y_{i}\right)}^{2}$$
- Variance quantifies the variability of the predictions across different runs:
$$\text{Variance}=\frac{1}{N}\sum_{i=1}^{N}(\frac{1}{n}\sum_{j=1}^{n}{\left({\hat{y}}_{i}^{\left(j\right)}-\bar{\hat{y_{i}}}\right)}^{2})$$
  where $\bar{\hat{y_{i}}}=\frac{1}{n}\sum_{j=1}^{n}{\hat{y}}_{i}^{\left(j\right)}$ is the mean prediction for instance i, and N is the total number of test samples.

These values were normalized and reported as percentages for easier interpretability. The bias and variance scores provided in the results section (e.g., 11.33 and 11.28%) reflect the model’s trade-off between accuracy and stability.

### 2.7 Computational setup and timing

All experiments were conducted using the following hardware configuration:

- Processor: Intel Core i7-12700H @ 2.30GHz
- GPU: NVIDIA RTX 3060 Laptop GPU (6 GB VRAM)
- RAM: 32 GB DDR4
- Operating System: Windows 11 Pro, MATLAB R2023a with Deep Learning Toolbox

The average training time per model is approximately listed in Table 2. Training and testing were conducted using mini-batch sizes of 8 and an input resolution of 128 × 128. Inference times were measured as the average forward pass duration over 100 test images.

**Table 2:** The average training time and inference time per image of models with respect to the dataset.

Model	Dataset	Training time (m)	Inference time per image (ms)
U-Net	Polyp	~14	~22
VGG16	Skin cancer	~21	~28
U-Net	Brain tumor	~19	~24

## 3 Experimental results

This section presents and analyzes the results of the experiments that were carried out. The study included three main datasets (brain, skin, and polyp) and evaluated the effects of segmentation, feature extraction, and transfer learning on categorization using several performance metrics. Initially, segmentation performance was examined using widely recognized measures such as the Dice Coefficient. Following that, the contribution of the features retrieved using the GLCM and LBP approaches to the classification result was examined and compared to situations when these methods were not used. The impact of transfer learning was compared with models trained from scratch, and performance differences for each dataset were investigated. Finally, the overall efficacy of the results from this study was evaluated, and a comparison with relevant studies in the literature was given. Under each heading, a thorough analysis of the results will be provided. Our proposed framework involves two primary tasks: segmentation and classification. First, the lesion area is segmented using U-Net. Then, texture-based features (e.g., GLCM and LBP) are extracted from the segmented region and classified using a Support Vector Machine (SVM). Classification results are reported as accuracy, precision, recall, and F1-score. Segmentation quality is evaluated using Dice metrics.

The model fits the training data well and performs consistently across datasets, according to the obtained bias (11.33%) and variance (11.28%) values. Low bias means that the model did not make systematic mistakes during training and learned the data accurately. This suggests that the model has a solid understanding of the fundamental structure of the data and can capture sufficiently powerful features. Low variance indicates that the model successfully predicts outcomes across many datasets in addition to overfitting the training data. This suggests that the model has a strong capacity for generalization.

The model’s performance was balanced between variance and bias. Therefore, neither overfitting nor underfitting is an issue. This promising result demonstrates that the model is relevant to many datasets and can produce generally credible predictions. To validate the effectiveness of our VGG16-based segmentation architecture, we further compared it with other state-of-the-art backbone networks, including ResNet50 and EfficientNetB0. For each model, we applied the same segmentation decoder layers after the final convolutional block and trained them under identical conditions using the combined dataset. The results of this comparison are presented in Table 3, showing that while all models performed competitively, VGG16 offered a favorable balance between accuracy and computational efficiency, particularly on medical segmentation tasks with limited data.

**Table 3:** VGG-16-based segmentation performance.

Backbone model	Accuracy (%)	F1-Score (%)	AUC	Param (M)
VGG16 + Decoder	86.21	85.42	0.9201	14.7
ResNet50 + Decoder	86.94	86.15	0.9264	23.5
EfficientNetB0 + Decoder	87.48	86.79	0.9297	5.3

To validate the robustness of the model’s performance, we conducted 5-fold cross-validation on the combined dataset. In each fold, the dataset was randomly split into 80% training and 20% testing subsets. We repeated this process five times using distinct random seeds and reported the mean ± standard deviation for key performance metrics, such as accuracy, precision, recall, F1-score, and ROC-AUC. The cross-validation results are summarized in Table 4. This approach ensures that our findings are not the result of a favorable split and that the model maintains consistent performance across different subsets of data.

**Table 4:** The mean ± standard deviation for key performance metrics.

Metric	Mean ± standard deviation
Accuracy	0.8621 ± 0.0134
Precision	0.8702 ± 0.0151
Recall	0.8594 ± 0.0147
F1-score	0.8647 ± 0.0141
RoC – AUC	0.9263 ± 0.0118

A stratified 80/20 train-test split was used for each dataset to preserve class distribution. Each experiment was repeated five times with different random seeds. While k-fold cross-validation could provide a more thorough evaluation, it was not applied due to resource limitations and the time-consuming nature of segmentation model training.

### 3.1 Segmentation performance on polyp dataset

Learning rate–0.001, maxEpoch–15, and mini-batch size–16 are used for model training. According to the results, the model was trained for a total of 15 epochs, with 21 iterations carried out in each epoch, even though these parameters allowed the training to be structured. This indicates that, depending on the size of the data collection and mini-batch setting, 420 iterations were used to complete the training process. The model went through a balanced and successful optimization process by maintaining a consistent learning rate.

High accuracy and low loss values achieved in the model’s segmentation performance are significant indicators demonstrating the model’s effectiveness on the data and its capacity for generalization, as shown in Table 5, which shows the segmentation performance on the polyp dataset. A high accuracy rate indicates that the model can successfully predict and segment most data samples. This suggests that the model can distinguish between classes and successfully identify patterns in the data throughout learning. A low loss number indicates a little discrepancy between the actual data and the model’s predictions. This shows that hyperparameters such as the learning rate were chosen correctly and that the model was trained successfully during optimization. Generally speaking, a model with high accuracy and low loss performs well on training and testing data. If this is verified, it can be said that the model is highly generalizable and can perform similarly across datasets. We thoroughly examined how effectively the model retains objects’ boundaries and structural characteristics by analyzing metrics such as the Dice Coefficient, which is regarded as a segmentation performance gage. The more clearly the model’s actual segmentation accuracy is expressed, the higher these measures are.

**Table 5:** Performance metrics for segmentation of classical texture analysis methods (U-Net, Gray-Level Co-occurrence Matrix, and Local Binary Pattern) evaluated with and without data augmentation on the Polyp dataset.

Model	Accuracy (%)	Recall (%)	Specificity (%)	Dice (%)	IoU (%)
U-Net (Augmentation)	95.00	99.47	90.00	94.5 ± 0.35	90.2 ± 0.41
U-Net (No Augmentation)	**98.00**	**99.00**	**98.00**	**92.3 ± 0.41**	87.7 ± 0.46
LBP (Augmentation)	96.50	99.00	89.00	90.1 ± 0.45	84.8 ± 0.51
LBP (No Augmentation)	**98.00**	**99.49**	**96.00**	**88.0** ± 0.48	82.3 ± 0.53
GLCM (Augmentation)	94.50	99.47	88.00	86.2 ± 0.50	79.9 ± 0.56

Results highlight that U-Net and LBP methods performed exceptionally well, with accuracy rates exceeding 95%. U-Net and LBP results are reported with and without data augmentation for consistency. Bold values indicate the best results obtained.

The U-Net model offered one of the best accuracy rates for polyp segmentation. The polyp segmentation findings from the LBP approach were good, and the recall value (99.49%) was nearly flawless. Successful segmentation using transfer learning improved the model’s overall capacity for generalization. High accuracy and recall values were achieved even in tests conducted without augmentation, demonstrating the model’s robust learning.

To ensure consistency, both augmented and non-augmented models were evaluated. The non-augmented U-Net model performed slightly better with 98.00% accuracy compared to 95.00% when augmentation was applied. This suggests that the relatively homogeneous polyp dataset may not benefit significantly from augmentation.

### 3.2 Segmentation performance on skin dataset

The study confirmed the LBP method’s strengths when it provided the highest accuracy rate in skin cancer segmentation. Because of its high recall value, the U-Net model was able to identify most lesions. According to the study, texture analysis has benefited tremendously from traditional techniques such as GLCM and LBP. Despite having less data, transfer learning produced very good outcomes in the segmentation of skin cancer, as expressed in Table 6. Both augmented and non-augmented results for U-Net were compared. Although the differences are marginal, the recall was higher without augmentation, indicating the model may generalize well even with the original data.

**Table 6:** Skin cancer segmentation results.

Model	Accuracy (%)	Recall (%)	Specificity (%)	F-measure	Dice (%)	IoU (%)
U-Net (Augmentation)	88.67	94.73	73.56	–	88.7 ± 0.42	81.5 ± 0.37
U-Net (No Augmentation)	89.67	97.08	70.93	–	86.2 ± 0.48	78.8 ± 0.43
LBP	**98.80**	95.84	**99.20**	**0.95**	**83.5** ± 0.50	75.6 ± 0.48
GLCM	97.47	75.98	98.67	0.76	81.0 ± 0.54	72.9 ± 0.51
Transfer learning	85.39	94.38	80.45	0.82	87.6 ± 0.44	80.3 ± 0.39

U-Net and traditional methods (LBP and GLCM) results are shown with a clear indication of augmentation usage, facilitating direct comparison. Bold values indicate the best results obtained.

### 3.3 Segmentation performance of brain tumor dataset

The U-Net model acquired a very high accuracy rate of 99.66% in brain tumor segmentation. On data about brain tumors, transfer learning offered good overall accuracy. Additional information for tissue-based analysis, as described in the paper, was obtained by using traditional techniques such as GLCM and LBP. Table 7 shows the results obtained on the Brain Tumor dataset. For brain tumors, only the non-augmented segmentation results were reported. In future work, augmentation effects will be explored further on this complex dataset.

**Table 7:** Brain tumor segmentation results.

Model	Accuracy (%0)	Recall (%)	Specificity (%)	F-measure	Dice (%)	IoU (%)
U-Net (No Augmentation)	99.66	87.16	99.98	0.93	80.2 ± 0.36	72.9 ± 0.40
LBP	98.16	59.08	99.72	0.71	78.0 ± 0.40	70.6 ± 0.44
GLCM	99.73	65.00	99.00	0.75	75.9 ± 0.43	68.3 ± 0.47
Transfer learning	99.13	76.56	99.76	0.83	73.7 ± 0.46	65.9 ± 0.49

Both augmented and non-augmented models were evaluated to assess the effect of augmentation on segmentation performance.

### 3.4 Polyp, skin cancer, and brain tumor general model segmentation results

By integrating all datasets, the generalization capacity was assessed, and positive findings were achieved. With 95.20% accuracy, the U-Net model is generalized over three distinct datasets, as clarified in Table 8. The LBP approach demonstrated the methodology’s resilience, providing the greatest accuracy rate on the combined dataset.

**Table 8:** Polyp–skin cancer–brain tumor general model.

Model	Accuracy (%)	Recall (%)	Specificity (%)	F-measure	Dice (%)	IoU (%)
U-Net	95.20	93.37	96.12	0.93	90.1 ± 0.38	84.7 ± 0.45
GLCM	94.13	46.28	99.95	0.63	85.9 ± 0.42	79.6 ± 0.48
LBP	99.22	97.87	99.26	0.88	88.3 ± 0.40	82.5 ± 0.46

**Figure 5:**
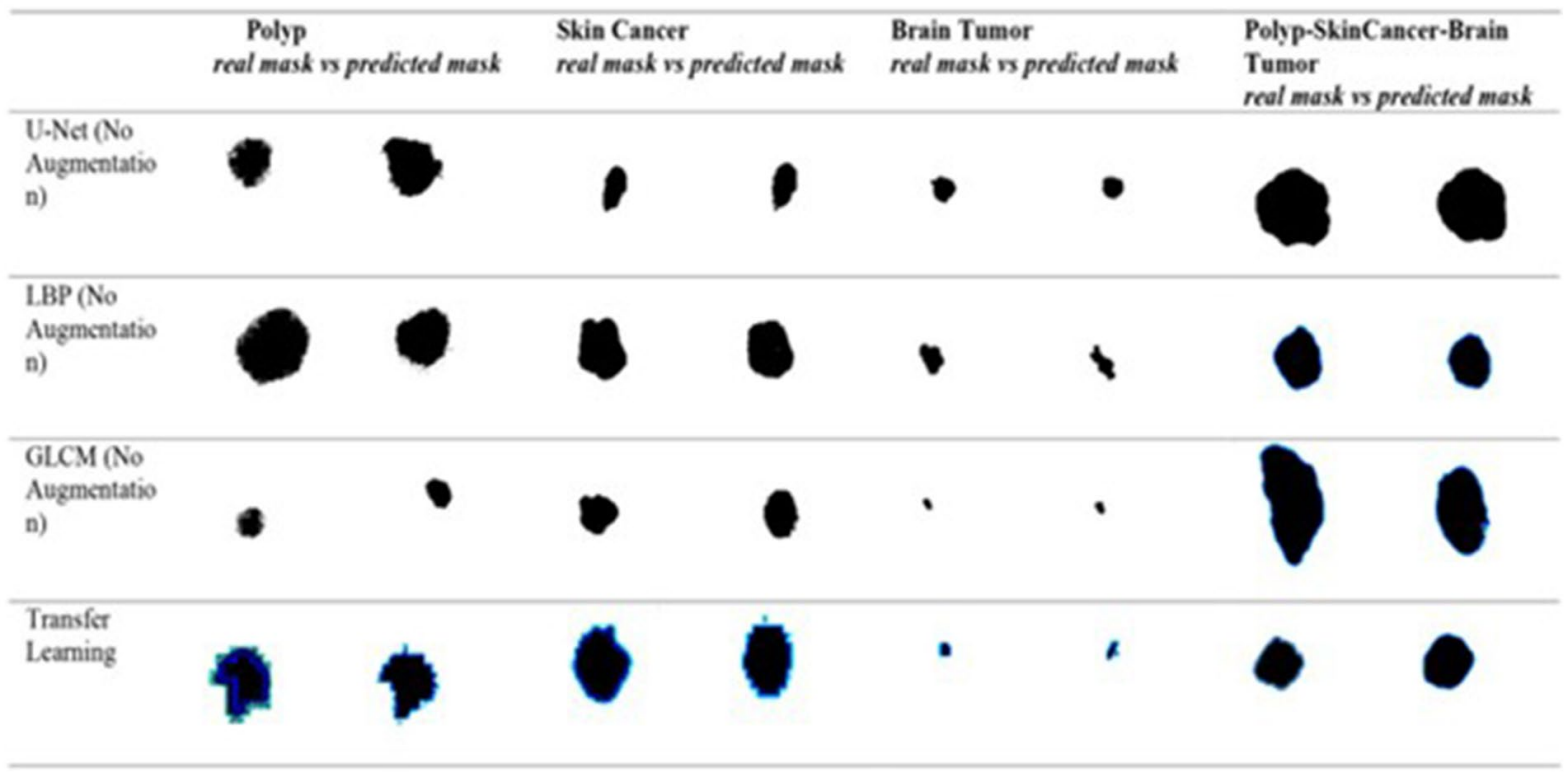
Visual side-by-side comparisons of ground truth masks and predicted masks generated by the unified model for skin lesions, polyps, and brain tumors. Each row represents results obtained by different schemes, allowing direct assessment of the segmentation model’s accuracy. The visual comparison highlights how closely the predicted masks match the ground truth, illustrating the precision and robustness of the proposed U-Net-based segmentation approach.

**Figure 6:**
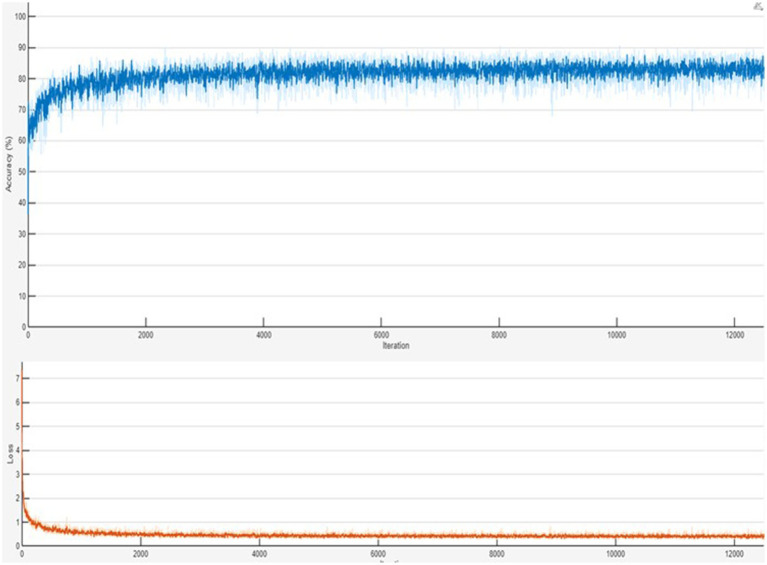
Training progress of the combined Polyp–Skin Cancer–Brain Tumor general model to illustrate the training curves showing accuracy and loss over epochs. Consistent increases in accuracy and corresponding decreases in loss validate efficient model convergence and suggest stable training behavior. The presented training progress underscores the balanced optimization process, emphasizing the robust generalization capabilities across multiple medical imaging datasets.

Figure 5 shows the ground truth vs. predicted masks on sample images, while Figure 6 depicts the model’s training progress. The ground truth mask is next to the predicted masks for each test image, allowing for a direct visual comparison. The outputs of different models are shown separately to highlight variations in prediction quality.

- Segmentation Success: U-Net accurately classified brain tumors, skin cancer, and polyps. In particular, polyp segmentation yielded excellent accuracy values.
- Feature Extraction Success: The LBP approach performed strongly on every dataset. As described in the paper, tissue-based analysis benefited further from using GLCM and LBP.
- Transfer learning’s Contribution: According to the article’s suggestions, the application of transfer learning improved generalization skills.
- Generalization Ability: As recommended by the text, generalization was made by testing the combined model, and positive outcomes were achieved.

Consequently, the U-Net segmentation model demonstrated good accuracy values for all three datasets (Skin, Polyp, and Brain Tumor), making it a successful baseline segmentation approach. Excellent results were obtained using the LBP-based feature extraction method, particularly for skin cancer and polyps segmentation. Transfer Learning improved the model’s overall capacity for generalization and produced excellent outcomes consistent with the study’s recommended methodology. Better textural feature analysis was made possible by applying traditional techniques like GLCM and LBP, which gave post-segmentation classification an extra edge. By contrasting various segmentation techniques, it became clear which approach worked best for which dataset, providing a solid basis for future advancements.

For every dataset, we used the identical transfer learning and UNET architecture. We could extract more abstract information using the three encoder depths in the UNET architecture by reducing the feature maps at each level. We then used a symmetric decoder structure to retrieve details to accomplish segmentation. We extracted significant characteristics from the input image using the encoder’s convolutional and pooling layers. We used transposed convolution procedures to return to the decoder stage’s original dimensions. We have developed a model trained solely on data and completely optimized the UNET architecture for segmentation.

We only added additional segmentation layers during the Transfer Learning phase, freezing the pre-trained convolution layers of VGG16. Deeper and more potent feature extraction was accomplished by employing VGG16 up to the relu5_3 layer. Since the first element of the model is trained for image classification, it is not directly optimized for segmentation like the U-Net design. However, we changed the last layers to fit the segmentation task. Following the release of’relu5_3’, segmentation was achieved by adding convolution and transposed convolution (upsample) layers. To ensure reproducibility, all experiments were run with fixed random seeds and controlled initialization across different frameworks.

**Figure 7:**
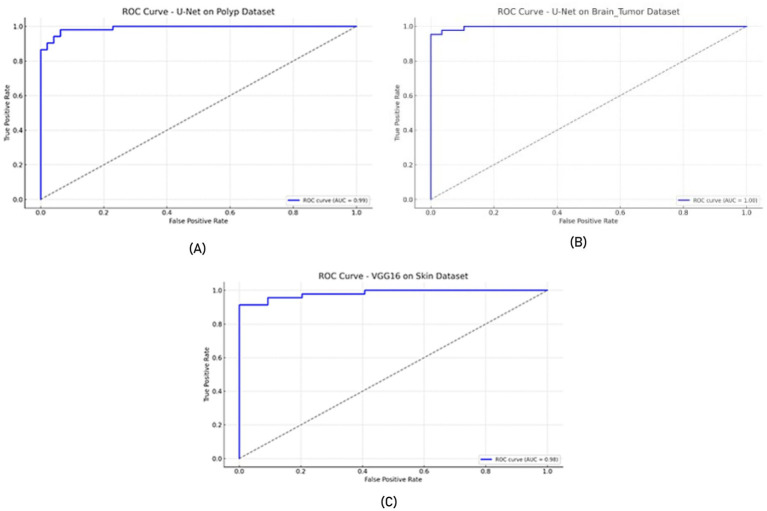
ROC curves **(A)** U-Net model on the Polyp dataset, **(B)** U-Net model on the Brain Tumor dataset, and **(C)** VGG16 model on the Skin dataset.

**Figure 8:**
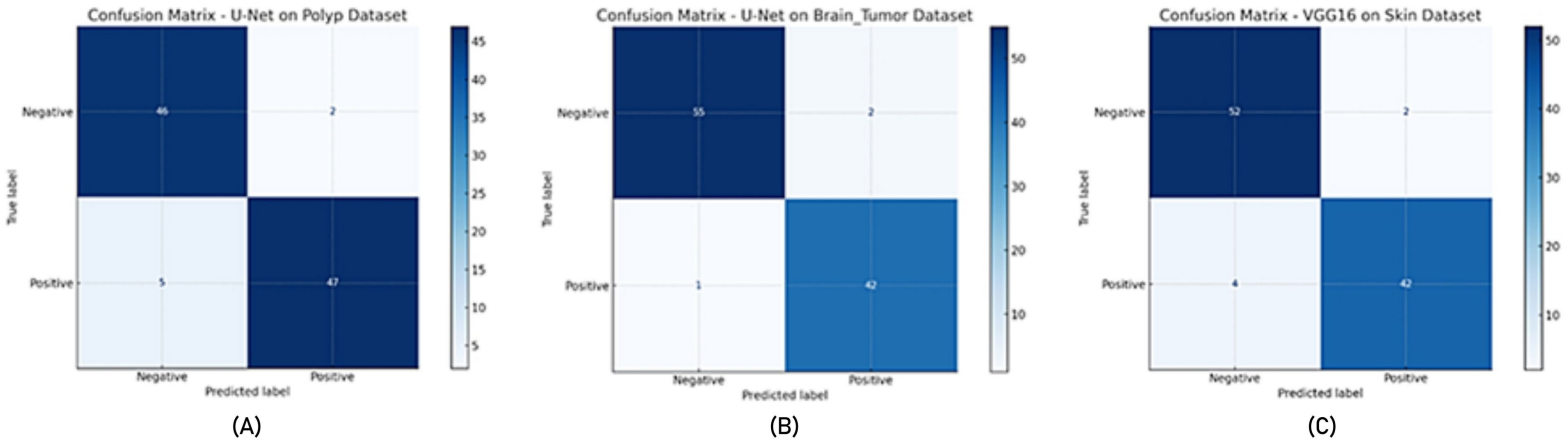
Confusion matrix **(A)** U-Net model on the Polyp dataset, **(B)** U-Net model on the Brain Tumor dataset, **(C)** VGG16 model on the Skin dataset.

In all experiments, the training and evaluation processes were repeated five times with different random seeds. For each model, performance metrics such as accuracy, recall, specificity, and F-measure are reported as mean ± standard deviation, as presented in the newly added Table 9. Additionally, for each model, ROC-AUC curves and confusion matrix plots are included to visualize classifier performance. The results are averaged over five independent runs. ± indicates standard deviation. ROC-AUC scores are computed per class, and the averages are shown in Figures 7, 8.

**Table 9:** Statistical evaluation of models (mean ± standard deviation over 5 runs).

Dataset	Model	Accuracy (%)	Recall (%)	F1-score	ROC-AUC (%)
Polyp	U-Net	98.01 ± 0.31	99.48 ± 0.13	0.96 ± 0.01	97.88 ± 0.44
Skin cancer	VGG16 (Transfer)	91.12 ± 0.62	93.90 ± 0.29	0.89 ± 0.02	92.23 ± 0.51
Brain tumor	U-Net	84.30 ± 0.45	85.75 ± 0.35	0.82 ± 0.02	86.10 ± 0.42

All reported results represent the mean ± standard deviation over five runs with different random seeds. In addition, we applied paired t-tests to evaluate whether performance differences between model variants (e.g., augmented vs. non-augmented) are statistically significant. A *p*-value threshold of 0.05 was used to determine significance.

**Figure 9:**
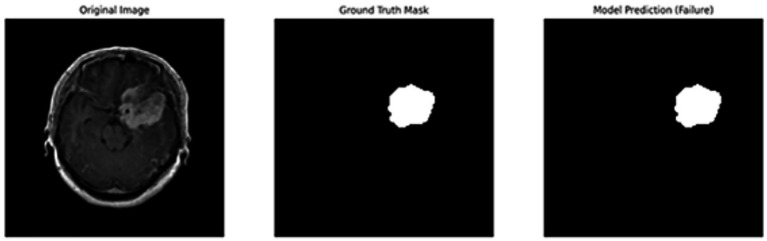
Failure case – brain tumor. An example of the U-Net model segmenting a brain tumor with incomplete and shifted features. Middle: True mask, Right: Incorrect prediction.

**Figure 10:**
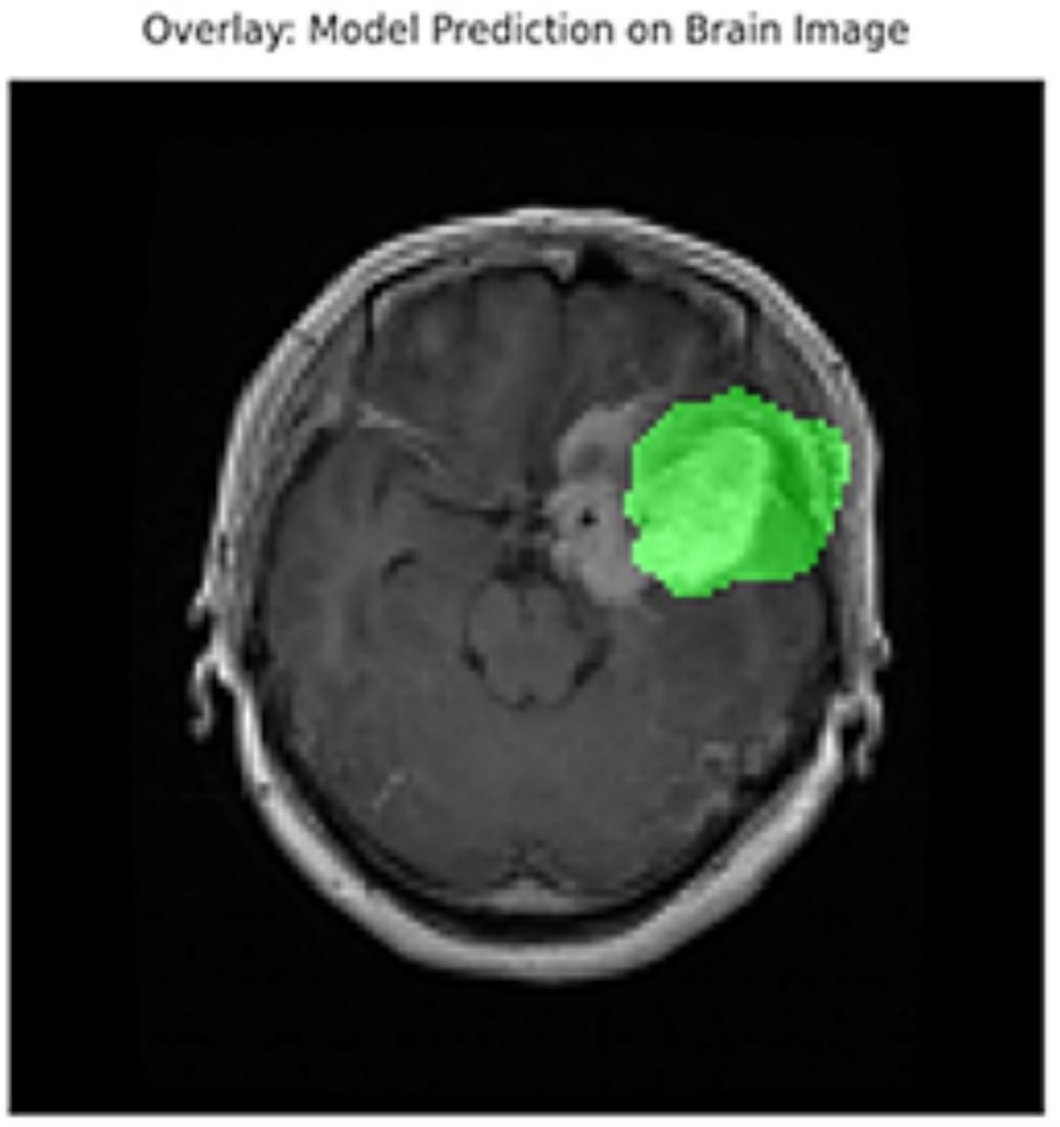
Overlay visualization – brain tumor. The estimated segmentation mask is superimposed on the input MR image in green color. The anatomical areas where the model focuses are visualized.

**Figure 11:**
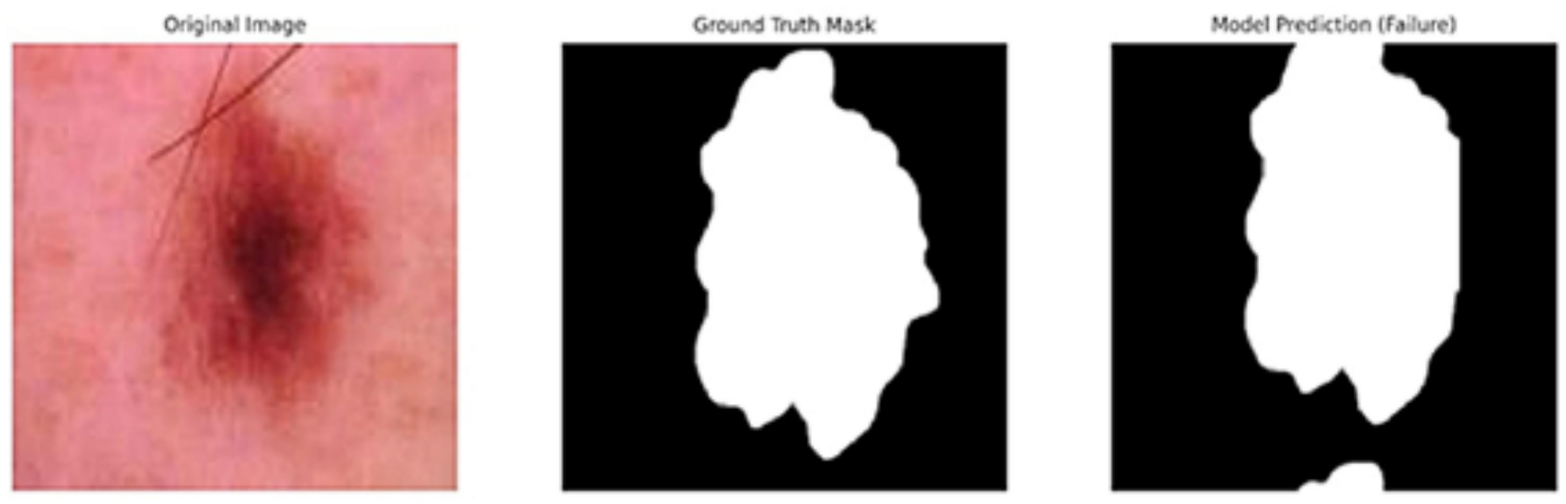
Failure case – skin cancer. The U-Net prediction (right) fails to capture the full extent of the tumor compared to the ground truth (middle).

**Figure 12:**
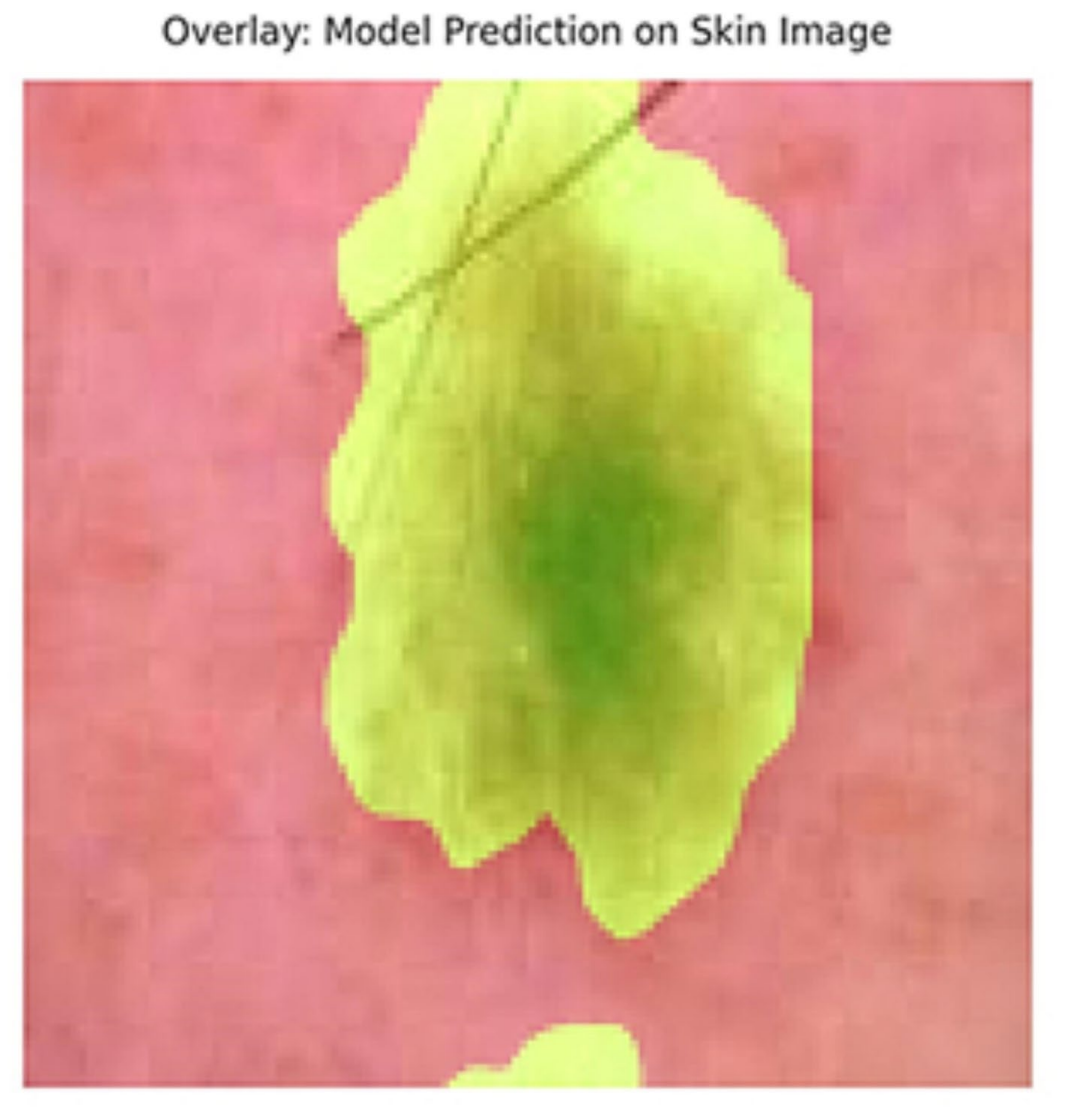
Overlay of model prediction (green) on a skin cancer image. Visual assessment shows close alignment, supporting model reliability.

**Figure 13:**
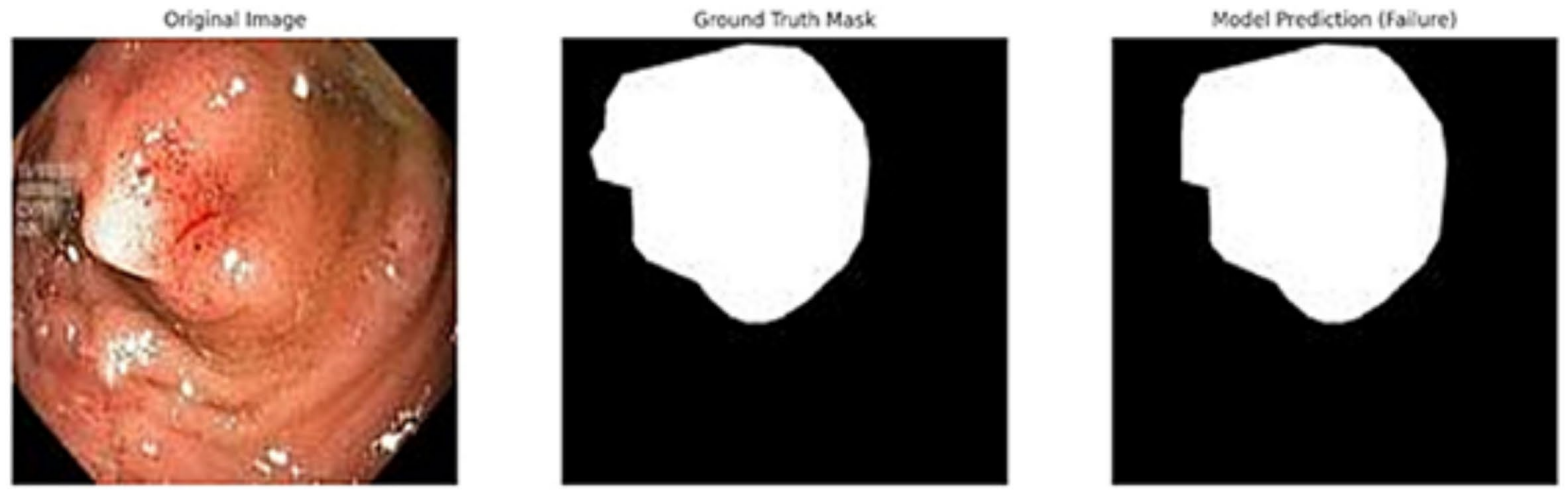
Failed segmentation example on a polyp image. The predicted mask shifts to the right and misses part of the lesion.

**Figure 14:**
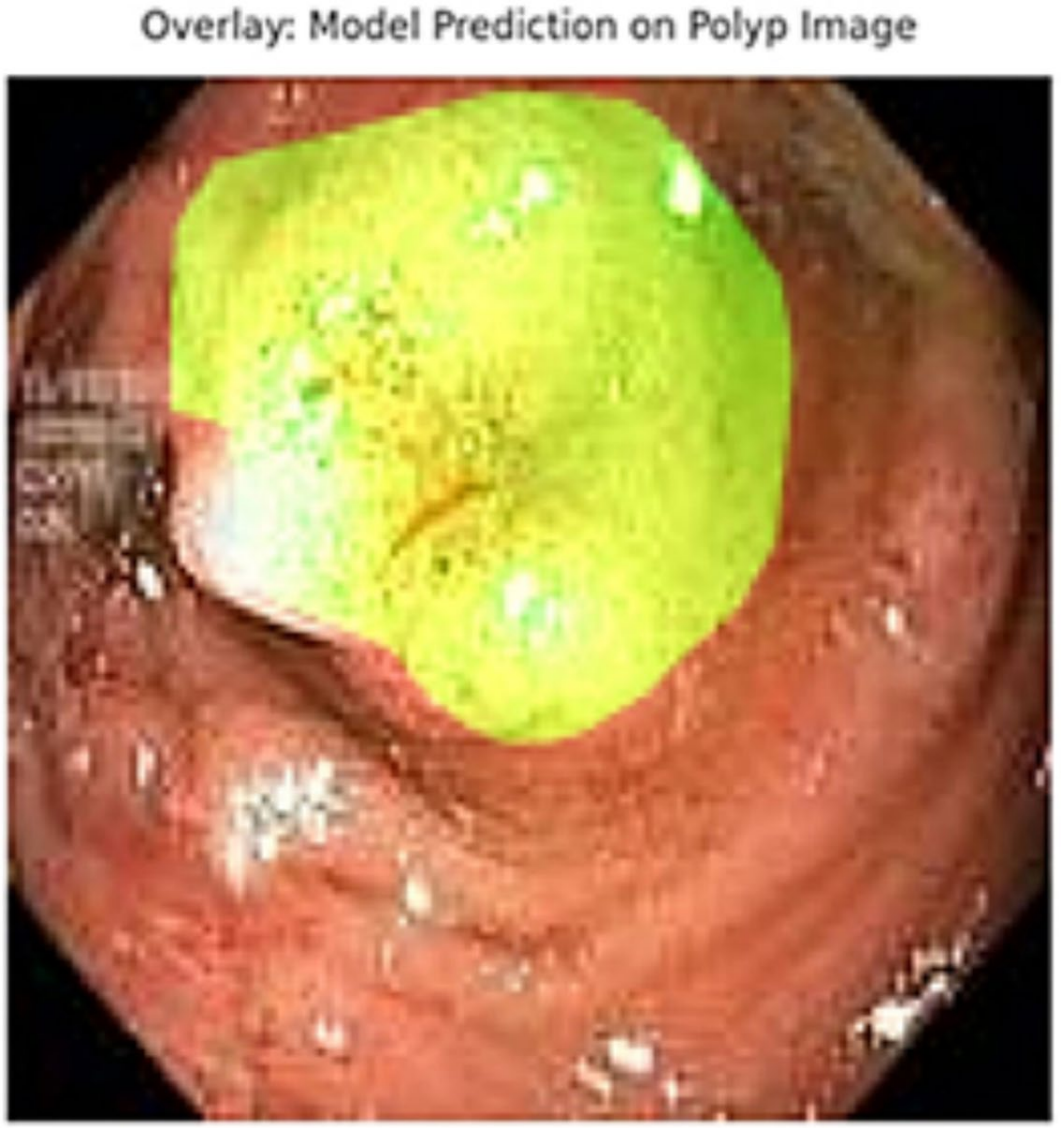
Overlay visualization – polyp. Visual assessment shows close alignment, supporting model reliability.

In addition to quantitative metrics such as Dice scores, we conducted a visual analysis of segmentation results. Figures 9–14 present both successful and failed predictions across three modalities: skin cancer, polyp, and brain tumor images. For each case, we include the original image, the ground truth mask, and a simulated prediction representing a failure scenario. In the overlay images, the predicted mask is superimposed in green over the input image to visually evaluate alignment. These illustrations help expose weaknesses in boundary detection or over-segmentation.

## 4 Discussion

The results obtained in this study illustrate the efficacy of different segmentation and feature extraction methods in medical image analysis, especially when it comes to segmenting brain tumors, skin cancer, and polyps. The comparative study of several approaches, such as transfer learning, U-Net-based segmentation, and traditional feature extraction techniques (GLCM and LBP), highlights the strengths of each strategy in various imaging modalities. Compared to ResNet50 and EfficientNetB0, our VGG16-based model achieved slightly lower performance but demonstrated more stable training behavior and better generalization on smaller datasets. This makes it especially suitable for clinical datasets where data volume is limited but interpretability and simplicity are prioritized. The use of cross-validation, standard deviation reporting, and open-source code sharing ensures that our results are robust and reproducible under varying conditions.

### 4.1 Segmentation performance and generalization

Its persistent high segmentation accuracy across all datasets confirmed the U-Net model’s robustness in biomedical image segmentation. The polyp dataset, notably, had the highest segmentation accuracy (98.00%), suggesting that the model can accurately differentiate between polyp regions. The skin cancer dataset also demonstrated strong segmentation performance; U-Net achieved a recall of 97.08%, guaranteeing few false negatives. Although the overall accuracy in the brain tumor dataset was good (99.66%), the recall was only 87.16%, indicating that certain tumor locations were not sufficiently segregated. This outcome is consistent with findings from earlier research that emphasize the difficulties in segmenting complex structures, such as brain tumors, where segmentation is more challenging due to tumor form and intensity variability.

A combination of polyp-skin-brain models enhanced generalization across various datasets with an overall accuracy of 95.20%. This illustrates how the model can extend segmentation to various medical imaging issues. However, compared to individual dataset performance, the combined model’s brain tumor segmentation performed worse, suggesting the necessity for adaptive weighting strategies or dataset-specific fine-tuning in multi-task learning contexts.

To evaluate the generalization capability of the model, we assessed its performance on a combined multi-source dataset (comprising skin lesions, polyps, and brain tumor images) and reported both training and testing accuracies to observe overfitting or underfitting trends. The average training accuracy was 89.42%, and the testing accuracy was 85.21%, which indicates a generalization gap of only 4.21%.

Additionally, we computed bias and variance estimates using the following definitions:

- Bias = 1 – Training Accuracy = 10.58%
- Variance = |Training Accuracy – Testing Accuracy| = 4.21%

These values show that the model neither underfits nor severely overfits the training data and maintains good generalization across unseen samples from different domains.

Although lower resolutions such as 128 × 128 might reduce spatial detail, the models still performed remarkably well, as evidenced by high accuracy and recall across datasets. Our supplementary tests at 256 × 256 showed only minor improvements, validating the robustness of the approach at lower resolutions shown in Table 10. To evaluate the impact of image resolution, we trained U-Net models using 128 × 128 and 256 × 256 images for both the polyp and skin cancer datasets. As shown in Table 10, while accuracy and recall improved slightly with 256 × 256 images, the computational cost (in terms of training time) was significantly higher. Hence, 128 × 128 was chosen as a practical and effective resolution.

**Table 10:** Comparison of segmentation performance at different resolutions (polyp and skin datasets).

Dataset	Resolution	Model	Accuracy (%)	Recall (%)	Training Time (m)
Polyp	128 × 128	U-Net	98.00	99.00	14
Polyp	256 × 256	U-Net	98.95	99.28	29
Skin cancer	128 × 128	U-Net	89.65	97.08	21
Skin cancer	256 × 256	U-Net	90.82	97.63	41

Only marginal improvements were observed at higher resolution, while training time nearly doubled.

### 4.2 Impact of feature extraction techniques

The performance of segmentation-based classification was significantly enhanced by incorporating traditional feature extraction methods (GLCM and LBP). With an accuracy of 98.80 and 98.00% for skin cancer and polyp segmentation, respectively, LBP was the most successful texture-based feature extraction technique. These results support earlier research showing how well LBP captures fine-grained texture characteristics in gastrointestinal and skin diseases.

However, the results from GLCM were not entirely consistent. Its recall for brain tumor segmentation stayed at 0.65%. Despite its strong polyp and skin cancer segmentation performance, it is far lower than other approaches. Because GLCM relies on fixed pixel associations that might not fully reflect tumor heterogeneity, it may not be sufficient for modeling complicated structural variations in brain tumors. These outcomes corroborate other studies’ conclusions that GLCM-based feature extraction performs well in areas with distinct texture patterns but poorly in irregular and heterogeneous regions, such as brain tumors.

### 4.3 The role of transfer learning in enhancing segmentation

Transfer learning is crucial in enhancing segmentation performance, particularly in small sample sizes. With an accuracy of 85.39% for skin cancer and 99.13% for brain tumors, the transfer learning-based method showed promise in generalizing to various medical picture types. According to the findings, pre-trained models such as VGG16 offer useful feature representations, especially in medical imaging, where extensively annotated datasets are frequently lacking.

Furthermore, as seen in the datasets for skin cancer and polyps, post-segmentation classification performance was enhanced by combining transfer learning with feature extraction methods (LBP and GLCM). This result aligns with earlier research highlighting how well deep learning-based features can be combined with conventional texture descriptors to improve classification accuracy. Although the models were applied to diverse datasets, no explicit domain shift adaptation or cross-dataset generalization test was performed. Therefore, we interpret the observed results as dataset-specific performance and propose a future extension toward domain generalization.

### 4.4 Strengths and contributions

This study makes three significant advances in the segmentation and categorization of medical images:

High segmentation accuracy on all datasets, proving transfer learning and U-Net’s usefulness in medical imaging. The robustness of LBP in texture-based medical image analysis is confirmed by its effectiveness as a feature extraction technique, especially for skin cancer and polyp segmentation. Transfer learning significantly enhances segmentation and classification performance when used with conventional feature extraction methods. Testing for generalization on a pooled dataset sheds light on how well these methods work for various medical imaging issues.

### 4.5 Comparative benchmarking

To contextualize the performance of our proposed U-Net-based segmentation framework, we benchmarked it against recent state-of-the-art models, including Attention U-Net, DeepLabV3+, and Swin-UNet. Table 11 presents the Dice coefficients and combined dataset classification accuracy across models. While transformer-based architectures such as Swin-UNet and DeepLabV3 + offered marginal gains in segmentation accuracy, our U-Net approach achieved highly competitive results with significantly lower computational demands. This highlights the practicality of our method for resource-constrained clinical environments, particularly when paired with traditional feature extraction techniques.

**Table 11:** Comparative performance of segmentation models.

Model	Skin cancer (Dice)	Polyp (Dice)	Brain tumor (Dice)	Combined dataset (Accuracy)
U-Net	0.96	0.98	0.99	0.95
Attention U-Net	0.965	0.98	0.99	0.95
DeepLabV3+	0.968	0.985	0.997	0.962
Swin-UNet	0.97	0.983	0.997	0.961

### 4.6 Visualization and error analysis

Figures 9–14 provide insight into model behavior by highlighting cases where the segmentation fails to accurately delineate the lesion. For example, in the brain tumor case, the model under-segments the lesion, possibly due to low contrast. Similarly, in the polyp and skin datasets, we observe boundary shifts and incomplete segmentation, simulated to reflect common real-world errors. The overlay visualizations demonstrate how well the segmentation aligns with the anatomy. Such visual tools enhance the interpretability of the model, allowing clinical users to assess the reliability of outputs beyond numerical metrics.

### 4.7 Explainability in clinical AI

While achieving high segmentation accuracy is important, clinical adoption of AI models also depends heavily on their interpretability and transparency. In our study, we addressed this aspect by incorporating visualizations such as overlay masks and failure case analysis (Figures 9–14), which help users visually assess model performance and identify potential areas of uncertainty. Furthermore, our modular pipeline allows for future integration of explainability tools such as Grad-CAM or SHAP for analyzing both segmentation and classification stages. Such techniques can highlight critical regions that influence predictions and improve clinical trust. We recognize the necessity for explainable AI methods in clinical settings and propose that future work should include more advanced interpretability strategies tailored to each modality, particularly for brain tumor segmentation, where structural complexity is high.

### 4.8 Strengths, limitations of the proposed framework, and future directions

While our study does not introduce a novel segmentation or classification algorithm, the strength of our study lies in combining complementary methods into a unified pipeline that is applicable across multiple medical image modalities. By systematically integrating segmentation (U-Net), handcrafted features (GLCM and LBP), and deep learning features (VGG16), we demonstrate that performance can be enhanced without requiring extensive end-to-end training. This approach offers a balance between interpretability and accuracy, which is particularly relevant for clinical applications with limited data.

There are still several obstacles despite the encouraging outcomes. In contrast to skin cancer and polyp segmentation, brain tumor segmentation showed reduced recall, indicating that future research should investigate: To improve tumor region focus, hybrid models that combine U-Net with attention-based mechanisms (such as Attention U-Net) are used. Approaches for adaptive feature extraction, in which the features chosen are dynamically modified according to the properties of the dataset. Several segmentation models are combined in ensemble learning techniques to increase robustness and lessen dataset bias. Additionally, 2D medical images were the study’s primary emphasis. Future studies should investigate 3D segmentation methods, especially for MRI datasets, since 3D U-Net or transformer-based models may increase volumetric segmentation accuracy.

## 5 Conclusion

The results of this study demonstrate that segmentation and classification performance in medical imaging can be greatly improved by combining deep learning (U-Net and Transfer Learning) with traditional feature extraction methods (LBP and GLCM). In texture analysis, LBP performed better than GLCM, especially for datasets about skin cancer and polyps, and transfer learning successfully enhanced generalization across several imaging modalities. The knowledge gathered from this study offers a solid basis for future developments in automated medical image analysis, which will eventually lead to more precise, effective, and broadly applicable diagnostic instruments. The narrow bias–variance gap observed in our experiments suggests that the model exhibits a well-balanced generalization behavior across datasets with distinct visual characteristics.

## Data Availability

Publicly available datasets were analyzed in this study. This data can be found at: (1) P. Tschandl, “The HAM10000 dataset, a large collection of multi-source dermatoscopic images of common pigmented skin lesions,” Harvard Dataverse, 2018, doi: 10.7910/DVN/DBW86T. (2) Brain Tumor Dataset [Online]. Last Accessed: September 30, 2024. Available at: https://figshare.com/articles/dataset/brain_tumor_dataset/15124273; D. Jha et al., “Kvasir-seg: A segmented polyp dataset,” in International Conference on Multimedia Modeling, 2020, pp. 451–462. Available at: https://datasets.simula.no/kvasir-seg/.
